# Illness unpredictability and psychosocial adjustment of adolescent and young adults impacted by parental cancer: the mediating role of unmet needs

**DOI:** 10.1007/s00520-021-06379-3

**Published:** 2021-07-09

**Authors:** Giulia Landi, Aylin Duzen, Pandora Patterson, Fiona E. J. McDonald, Elisabetta Crocetti, Silvana Grandi, Eliana Tossani

**Affiliations:** 1 Department of Psychology, University of Bologna, Viale Berti Pichat 5, 40127 Bologna, Italy; 2 Laboratory of Psychosomatics and Clinimetrics, Department of Psychology, University of Bologna, Viale Europa 115, 47023 Cesena, Italy; 3 Research, Evaluation and Social Policy, CanTeen Australia, M: GPO Box 3821, Sydney, NSW 2001 Australia; 4 Faculty of Medicine and Health, The University of Sydney, Missenden Road, PO Box M30, Sydney, NSW 2050 Australia

**Keywords:** Adolescents and young adults, Health-related quality of life, Illness unpredictability, Internalizing problems, Offspring unmet needs, Parental cancer

## Abstract

**Purpose:**

Given the large number of adolescents and young adults (AYAs) impacted by parental cancer and the potential for negative psychosocial outcomes in this vulnerable population, this study examined the mediating role of offspring unmet needs with regard to parental cancer and the relation between AYAs psychosocial adjustment and perceived illness unpredictability.

**Methods:**

A total of 113 AYAs (aged 11–24 years) living with a parent diagnosed with cancer completed a questionnaire assessing illness unpredictability, offspring unmet needs, and psychosocial adjustment (i.e., health-related quality of life and internalizing problems).

**Results:**

Higher offspring unmet needs were associated with lower health-related quality of life (*r* = –0.24**) and higher internalizing problems (*r* = 0.21*). Offspring unmet needs mediated the relation between illness unpredictability and health-related quality of life (standardized indirect effect = –0.100* [–0.183, –0.018]) but not internalizing problems (standardized indirect effect = 0.067 [–0.015, 0.148]). In particular, higher illness unpredictability was related to higher unmet needs (*β* = 0.351**) which, in turn, predicted lower health-related quality of life (*β* = –0.286**).

**Conclusion:**

These findings identify offspring unmet needs and illness unpredictability as implicated in AYAs positive psychosocial adjustment to parental cancer. Given that AYAs are at greater risk of elevated psychosocial difficulties, interventions should target offspring unmet needs and perception of illness unpredictability to mitigate the adverse effects of parental cancer.

In 2018, around 2–2.2 million cancer cases were diagnosed worldwide [1]. About 45% of these people are aged between 20 and 64 years; therefore, childbearing and parenting years are widespread periods to receive a parental cancer diagnosis [2]. Given that cancer is the second leading cause of death worldwide with high rates of new cases increasing each year, the number of offspring affected by parental cancer is likely to steadily rise [3–5]. Furthermore, worldwide, the prevalence of offspring affected by parental cancer is increasing, not only because cancer survival rates have increased by about 20% rise in the last 5 years [6], but also because parents are conceiving children at an older age. In turn, older parental age may be associated with raises in the number of offspring impacted by parental cancer [5].

A Lancet commission further identified the age range 11–24 years as the highest priority target for youth health and well-being research [7], and studies show that having a parent with cancer during this period can adversely affect the psychosocial adjustment of adolescents and young adults (AYAs) [8–12]. In comparison with their peers with “healthy” parents, AYAs living with a parent diagnosed with cancer are at increased risk of internalizing (e.g., depressive symptoms, anxiety and somatic symptoms) and externalizing problems (e.g., aggressive and delinquent behaviors) [8–10, 13–16], poorer quality of life, and lower life satisfaction [8–12]. Parental cancer is also associated with higher emotional dysregulation, stress-related somatic disorders, lower school performance, and higher unemployment rates in offspring [9, 10, 12]. According to one longitudinal study, AYAs experiencing parental cancer consulted psychiatric services with greater frequency and at an earlier age than offspring of “healthy” parents [17].

Another indicator associated with increased anxiety and depression in family members affected by parental cancer is their levels of cancer related unmet needs [18], with higher unmet needs related to increased anxiety and depression [18–22]. The Offspring Cancer Needs Instrument (OCNI) [21] is a self-report measure that specifically assesses the unmet psychosocial needs of AYAs who have a parent with cancer. It is divided into seven areas of unmet needs (see Table 1 for OCNI domain descriptions). Higher unmet needs were reported in older offspring and in families characterized by poorer family functioning, as well as when parental cancer treatment was current or a relapse had occurred [20]. Furthermore, the need for information regarding parental cancer diagnosis, treatment implications, and prognosis has been highlighted as the strongest psychosocial need reported by AYAs [22, 23].

**Table 1:** Unmet needs in adolescents and young adults (AYAs) impacted by parental cancer

Dimensions of the Offspring Cancer Needs Instrument (OCNI)	
Information about my parent’s cancer	- This domain refers to offspring having access to information about their parent’s cancer and the need for conveying this information in a way understandable to them (e.g., “to get information about my parent’s cancer in a way that I can understand”)
Family issues	- This domain refers to the need for offspring to feel supported by their families and to communicate openly and honestly with them about parental cancer (e.g., “to feel that I can talk openly with my family about my parent’s cancer”)
Practical assistance	- This domain refers to the need for information and assistance with the caring of the ill parent and household duties as well as practical support for staying on tasks at school or work. It also includes having access to professional support services (e.g., “assistance with looking after my parent with cancer”)
“Time out” and recreation	- This domain refers the need for offspring to be involved in sport and social activities along with the need for occasional escapism and “time out” from the pressures of having a parent with cancer (e.g., “need to be able to have fun”)
Dealing with feelings	- This domain refers to the need for offspring to be able to express how they are feeling about their parent’s cancer along with the need for help when dealing with these feelings (e.g., “help dealing with feelings of anxiety and feeling scared about my parent’s cancer”)
Support from my friends	- This domain refers to the need to feel supported by one’s own friendship group who share a similar experience about having a parent with cancer (e.g., “my friends to understand what I am going through”)
Support from other young people	- This domain refers to the need to be supported by other young people who share a similar experience about having a parent with cancer (e.g., “to talk to someone my own age who has been through a similar experience with cancer”)

Note: Adapted from Patterson et al. (2013)

In addition, compared to objective indicators of severity of parental cancer (e.g., stage or prognosis), higher perceived parental cancer severity is associated with increased distress in AYAs [24]. One qualitative study corroborated these findings indicating that AYAs who perceived their parental cancer with greatest feelings of fear, uncertainty, and loss of control toward the illness experienced the highest negative emotional reactions [25]. Also, AYAs reported more anxiety, depression, and stress compared to a group of pre-adolescents impacted by parental cancer [24, 26]. AYAs living in families impacted by parental cancer may be particularly vulnerable to the negative psychosocial consequences of illness unpredictability (i.e., fear of recurrence, unpredictable symptoms and side effects), as they may be more aware of the seriousness and consequences of their parental cancer diagnosis but may not yet have developed their ability to cope with this unpredictable condition [27–29].

In line with previous research pointing to unmet needs as relevant predictors of psychosocial adjustment in AYAs [22, 25], the primary focus of this study was to further disentangle the relationship between offspring unmet needs and their psychosocial adjustment. In particular, we expanded on previous findings by further examining the mutual relationship of illness unpredictability and unmet needs and their link with positive (i.e., health-related quality of life) and negative (i.e., internalizing problems) outcomes in offspring of parents with cancer. We supposed that unmet needs would be a mechanism through which illness unpredictability exerts its effect on AYAs psychosocial adjustment. This prediction is line with one of the most promising model of youth adjustment in the context parental illness, the family ecology framework (FEF), which indicates that parental illness severity affects youth adjustment indirectly through various youth responses to parental illness [30]. Therefore, the aims of this study were as follows: (1a) to analyze the relationship between levels of unmet needs in AYAs and their psychosocial adjustment (i.e., health-related quality of life and internalizing problems) as well as (1b) to explore the impact of demographic (offspring age and gender, and parental gender) and cancer variables (time since diagnosis and illness unpredictability) on level of offspring unmet needs and (2) to investigate the mediating role of unmet needs in the relationship between illness unpredictability and psychosocial adjustment in AYAs. We expected that (H1a) higher unmet needs would be associated with worse psychosocial adjustment (i.e., poorer health-related quality of life and greater internalizing problems) and (H1b) higher levels of illness unpredictability, which is one of the cancer variables, would be related to higher levels of unmet needs; (H2) illness unpredictability would increase unmet needs which, in turn, would be associated with worse psychosocial adjustment in AYAs.

## Method

### Participants and recruitment procedure

We conducted this study in Italy, where it is estimated that about 3.5 million people are currently living with an oncological disease, with more than 1,000 new cases of cancer diagnosed every day [31]. Eligibility criteria included AYAs aged between 11 and 24 living with a parent affected by cancer. Exclusion criteria were insufficient command of Italian, cognitive impairments, and severe medical conditions in youth themselves, siblings, or other family members apart from parents. Data was collected between November 2018 and May 2019. Because there is a lack of official statistics describing the characteristics of Italian AYAs of parents with cancer, we could not directly compare the characteristics of our sample with the characteristics of the overall population of youth in the context of parental cancer. Nevertheless, in order to collect a sample that could be as representative as possible, we used multiple recruitment strategies such as information brochures and posters in primary and secondary schools, universities, and groups of youth (e.g., library, music, and sports groups); cancer-related local community organizations (e.g., self-help and family support groups); and waiting rooms of health facilities (i.e., general practitioner, hospital, and cancer specialist clinics). Potential participants who showed interest in taking part in the study contacted the researchers by telephone or email. Subsequently, a researcher conducted an initial interview collecting some socio-demographic and qualitative data and then gave AYAs a booklet of questionnaires to fill out, usually at the family home, after obtaining active informed consent from both parents or legal guardian if youth were under the age of 18 years or from youth themselves if they were ≥ 18 years. The administration procedure was pencil and paper-based. The variation in recruitment methods precluded the calculation of an overall response rate. The study was approved by the University of Bologna ethics committee.

### Measures

#### Demographics and family structure variables

AYAs indicated their age (via the date of birth), gender, studying status, and employment status. AYAs also indicated the number of family members, including siblings as well as whether they were living in a dual or single-parent family.

#### Parental cancer variables

AYAs indicated which parent had cancer (mother, father, or both) as well as the type of parental cancer. If both were selected, participants were asked to answer the remaining questions concerning the parent with the more severe type. Participants also indicated the time in years since their parent’s cancer diagnosis.

#### Perceived illness unpredictability

Perceived illness unpredictability was evaluated with a scale developed by Pakenham and colleagues [29] and used in prior published research in the field of youth caregiving (e.g., 28,29). AYAs indicated the extent to which they agreed with 5 items (e.g., “My parent’s condition could change at any time with little warning” or “It’s not clear to me when my *parent’s* condition is getting better or worse” or “It is difficult to plan ahead because my *parent’s* condition is unpredictable”) rated on a 5-point scale (0 strongly disagree to 4 strongly agree) [29]. Items scores were averaged with higher scores indicating higher illness unpredictability [29]. The internal consistency (α = 0.68) in the current sample was adequate.

#### Offspring unmet needs

The Offspring Cancer Needs Instrument (OCNI) [21] assesses the unmet psychosocial needs of offspring who have a parent with cancer. It is composed of 47 items clustered into 7 domains. Scores are summed, with higher scores indicating higher offspring unmet needs. The OCNI has evinced good psychometric properties [21]. A multistep approach was selected for the translation of OCNI into Italian [32]. The original version of the instrument was firstly independently translated by two authors and a bilingual translator. Ambiguities of these versions were identified, and a reconciled forward version was created. This preliminary version was back-translated by one bilingual translator whose native language was English. This back-translated version was submitted to the original authors for approval. After applying a few suggested changes, the Italian version of the OCNI was administered to a pilot group of 30 offspring to evaluate the extent to which the instrument was clear and understandable. Final modifications were carried out according to this pilot study. Because the OCNI has not been validated in Italian, as a preliminary step, we ran a confirmatory factor analysis (CFA) of the translated version. The final Italian OCNI used in this study demonstrated excellent internal consistency (α = 0.95).

#### Psychosocial adjustment

The following positive and negative psychosocial adjustment outcomes were assessed in AYAs: health-related quality of life and internalizing problems.

##### Health-related quality of life

The Kidscreen-27 [33] is a self-report questionnaire composed of 27 items assessing youth quality of life in five subscales: physical well-being, psychological well-being, autonomy and parent relations, peers and social support, and school environment. Total scores were computed by summing all items, with higher scores indicating greater health-related quality of life. Kidscreen-27 has been validated in a large population-based youth sample from several European countries, including Italy, and demonstrated good psychometric properties [33]. The internal consistency of Kidscreen-27 in this study was excellent (α = 0.82).

##### Internalizing problems

The internalizing problem scale of the youth self-report (YSR) was used to assess emotional and behavioral functioning of AYAs [34]. The YSR internalizing problems scale reflects three dimensions: anxious/depressed, withdrawn/depressed scale, and somatic complaints. Items are summed to obtain a total score for internalizing. The original YSR has demonstrated sound psychometric proprieties including test–retest reliability (*r* = 0.79 to 0.88), internal consistency (*r* = 0.67 to 0.83) and good content, and criterion-related and construct validity [34]. The Italian version of YSR has been validated and showed good psychometric properties [35]. The internal consistency (α = 0.88) in the current sample was excellent.

#### Data analysis

Data analyses were performed using statistical software SPSS and *Mplus* 8.3 [36]. Multiple linear regression was used to examine the impact of demographic and cancer variables on the level of offspring unmet needs. Cronbach’s alpha was used to estimate the internal reliability of all measures, with values greater than 0.60 considered to be acceptable, more significant than 0.70 satisfactory, and above 0.80 excellent [37].

The structural validity of the Italian translation of the OCNI was tested by confirmatory factor analyses (CFA) with maximum likelihood robust (MLR) estimator. To estimate the structural associations between the study variables, structural equation modelling (SEM) analyses were conducted with MLR. In particular, we tested models including a combination of latent (i.e., unmet needs, health-related quality of life, internalizing problems) and observed (i.e., illness unpredictability) variables. Demographics significantly correlated with youth adjustment outcomes were controlled for in the mediation analyses. Finally, to test the mediating effects of offspring unmet needs in the relationship between parental cancer unpredictability and psychosocial adjustment, indirect effects were tested. The model fit was evaluated by means of the comparative fit index (CFI), with values higher than 0.90 indicative of an acceptable fit, and values higher than 0.95 demonstrating an excellent fit, and the standardized root mean square residual (SRMR) and the root mean square error of approximation (RMSEA), with values below 0.08 representing an acceptable fit and values lower than 0.05 indicative of a very good fit [38]. The 90% confidence interval (CI) of the RMSEA was also examined (i.e., a good fit is indicated by the upper bound lower than 0.10) [38].

## Results

### Participants’ characteristics

One hundred and thirteen AYAs impacted by parental cancer took part in the study. The participants ranged in age from 11 to 24 years (*M* = 17.97, *SD* = 3.79), with 61.1% female and an average parental time since diagnosis of 3.07 years (*SD* 2.98), ranging from just diagnosed to 5 years since diagnosis. Further demographic characteristics of young people and their parents with cancer are depicted in Table 2.

**Table 2:** Demographic characteristics of the adolescents and young adults (AYAs) and their parents with cancer

Variable	%	M (SD)	Range
Demographics			
Age years		17.97 (3.79)	11.59–24.79
Gender			
Male	38.9		
Female	61.1		
Currently studying	87.6		
Currently working	28.3		
Family structure variables			
Family size		4.19 (1.21)	
Number of siblings		1.01 (0.07)	
Single-parent family	8.8		
Parent with cancer			
Mother	87.6		
Father	12.4		
Time since cancer diagnosis (years)		3.07 (2.98)	1–5
Illness unpredictability		1.6 (0.8)	0–3.6
Cancer types^1^			
Breast cancer	66.4		
Colon cancer	12.4		
Skin cancer	4.4		
Brain cancer	2.7		
Uterine cancer	2.7		
Thyroid cancer	1.8		
Kidney cancer	1.8		
Soft tissues tumor	1.8		
Lymphoma cancer	1.8		
Ovarian cancer	0.9		
Bone cancer	0.9		
Liver cancer	0.9		
Laryngeal cancer	0.9		
Auditory nerve tumor	0.9		

Notes: ^1^Some people had more than one type of cancer

### Preliminary analyses

#### Factor analysis of Italian Offspring Cancer Needs Instrument

**Fig. 1:**
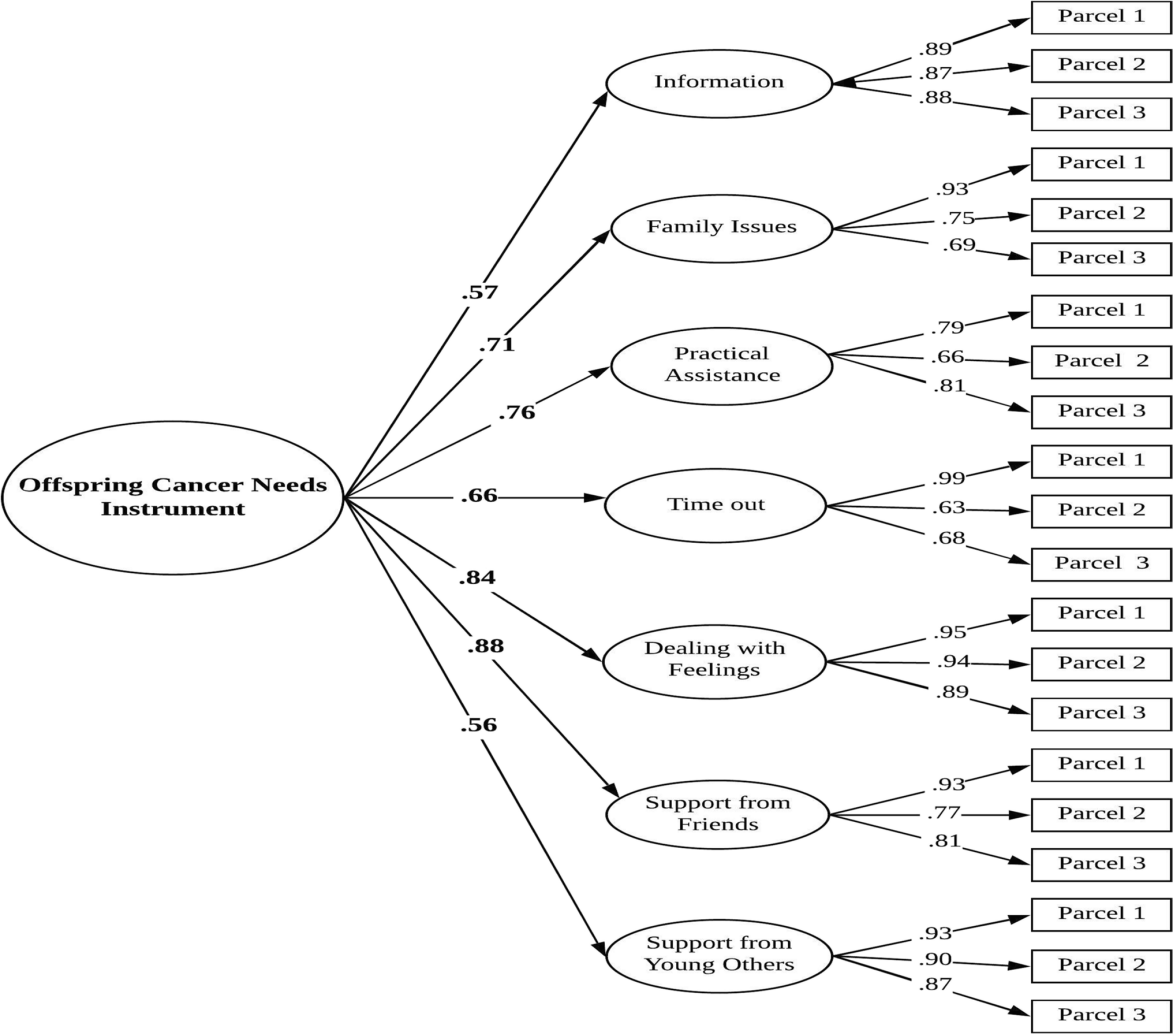
Standardized solution of the original factor model of the Italian Offspring Cancer Needs Instrument (OCNI). Note: All factor loadings are significant at p < 0.001

To test the original factor structure of the OCNI [21], a CFA was conducted using MLR estimator. Because there was variability in the number of items forming each of the OCNI dimensions (ranging from four to thirteen items), we used a parceling approach (random assignment of items to parcels) [39]. In this condition, the parceling technique has several advantages, such as a more optimal sample size ratio indication and a greater likelihood of achieving a good model solution [40, 41]. Specifically, we used three parcels for each of the OCNI dimensions, as reported in Fig. 1. Fit indices indicated that the original factor structure of the OCNI, with seven first-order latent factors and one second-order latent factor (i.e., total score of OCNI), fit the data very well also in this Italian sample (*χ*^2^ = 1828.745, *df* = 210, CFI = 0.925, RMSEA [90% CI] = 0.077 [0.061, 0.092], SRMR = 0.065). Cronbach’s alpha for each subscale were good (information about my parent’s cancer = 0.92, family issues = 0.82, practical assistance = 0.79, “time out” and recreation = 0.82, dealing with feelings = 0.92, support from my friends = 0.88, support from other young people = 0.94). Figure 1 displays standardized solution for the Italian OCNI factor structure.

#### Levels of unmet needs and AYAs psychosocial adjustment and the relationship between demographic and cancer variables on level of unmet needs

In line with the first study aim, we conducted Pearson’s correlations to analyze the relationship between levels of unmet needs in AYAs impacted by parental cancer and their psychosocial adjustment (operationalized with health-related quality of life and internalizing problems). As reported in Table 3, results indicate that higher unmet needs were negatively related to health-related quality of life (*r* = –0.24, *p* < 0.001) and positively related to internalizing problems (*r* = 0.21, *p* < 0.05). Illness unpredictability was positively associated with offspring unmet needs (*r* = 0.35, *p* < 0.01) and with internalizing problems (*r* = 0.22, *p* < 0.05), but was not associated with health-related quality of life. Health-related quality of life was negatively and strongly correlated with internalizing problems (*r* =  −0.60, *p* < 0.01). All study variables were normally distributed, and their means, standard deviations, skewness, kurtosis, and bivariate correlations are displayed in Table 3.

**Table 3:** Means (M), standard deviations (SD), skewness, kurtosis, and bivariate correlations among study variables

	M*(SD)*	Skewness, kurtosis	1	2	3
1. Offspring unmet needs	104.76 (26.84)	–0.15, –0.81	-		
2. Illness unpredictability	1.60 (0.80)	0.10, –0.52	0.35^**^		
3. Internalizing problems	13.93 (9.13)	0.85, 0.32	0.21^*^	0.22^*^	
4. Health-related quality of life	97.14 (15.38)	–0.51, –0.18	−0.25^**^	−0.14	−0.60^**^

Notes: *N* = 113. ^∗^*p* < 0.05, ^∗∗^*p* < 0.01, ^∗∗∗^*p* < 0.001

We further explored the impact of demographic variables (offspring age and gender, and parental gender) and cancer variables (time since diagnosis and illness unpredictability) on the level of offspring unmet needs. We fitted a multiple linear regression model with total unmet needs as the dependent variable and demographic and cancer variables as predictors. As shown in Table 4, among all included predictors, only illness unpredictability (*β* = 0.333, *p* < 0.01) was significantly positively associated with unmet needs.^1^ As expected, illness unpredictability was positively related to unmet needs in AYAs. The total model accounted for 15% of the variance in offspring unmet needs which represents a medium effect size [42].

**Table 4:** Linear regression examining the impact of demographic and cancer variables on the level of offspring unmet needs

	Offspring Unmet Needs	
	Coeff. (*SE*)	95% CI
Offspring age	0.006 (0.656)	−1.259, 1.343
Offspring gender	−0.011 (5.052)	−10.601, 9.434
Parental gender	0.095 (7.310)	−6.838, 22.149
Time since diagnosis	0.122 (0.820)	−0.534, 2.716
Illness unpredictability	0.333^∗∗^ (3.180)	4.952, 17.564
	R^2^ = 0.153^∗∗^ *F*(5,105) = 3.805	

Notes: *N* = 113*.* ^∗^*p* < 0.05, ^∗∗^*p* < 0.01, ^∗∗∗^*p* < 0.001. *Coeff.* standardized coefficient; *SE* standard error; *95% CI* 95% confidence interval

#### The mediating role of offspring unmet needs in the relationship between illness unpredictability and offspring psychosocial adjustment

In line with the second study aim, to examine the mediating role of offspring unmet needs in the relationship between illness unpredictability and offspring psychosocial adjustment, we conducted two SEM analyses in which illness unpredictability was set as the independent variable, offspring unmet needs as the mediator, and internalizing problems and health-related quality of life as dependent variables, respectively. Of the demographics, gender and age were significantly associated with internalizing problems and health-related quality of life; therefore, we controlled for them in mediation analyses. Specifically, being female positively related to internalizing problems (*r* = – 0.30, *p* < 0.01), while being older was associated with poorer health-related quality of life (*r* = – 0.21, *p* < 0.001). The two models fit the data well: *χ*^2^ = 113.724, *df* = 83, CFI = 0.941, RMSEA [90% CI] = 0.057 [0.027, 0.082], and SRMR = 0.060 for the model with health-related quality of life and *χ*^2^ = 87.781, *df* = 58, CFI = 0.935, RMSEA [90% CI] = 0.067 [0.036, 0.095], and SRMR = 0.063 for the model with internalizing problems as outcomes.

**Fig. 2:**
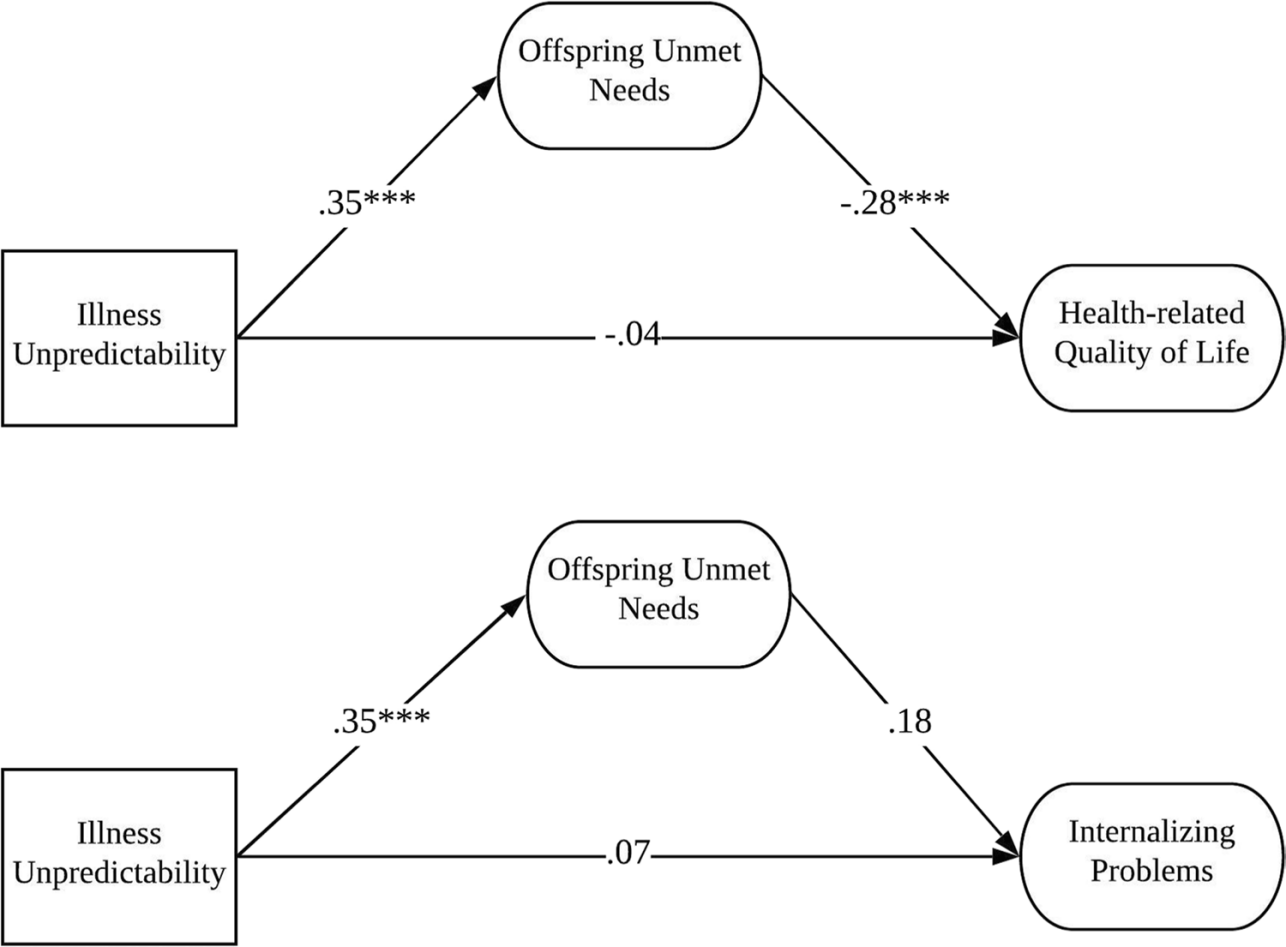
Mediating role of offspring unmet needs in the relation between AYA perception of illness unpredictability and both health-related quality of life and internalizing problems. Note: Analyses were conducted controlling for gender and age of AYAs

Results of the mediation analyses are displayed in Fig. 2. Illness unpredictability had no direct effect on health-related quality of life (*β* =  −0.048, *p* > 0.05) nor internalizing problems (*β* = 0.071, *p* > 0.05). As expected, offspring unmet needs significantly mediated the relationship between illness unpredictability and health-related quality of life: standardized indirect effect = –0.100 [–0.183, –0.018], *p* = 0.017. In particular, illness unpredictability was positively associated with offspring unmet needs (*β* = 0.351, *p* < 0.001), which in turn were negatively related to health-related quality of life (*β* = –0.286, *p* < 0.01). However, contrary to our hypothesis, offspring unmet needs did not significantly mediate the relationship between illness unpredictability and internalizing problems: standardized indirect effect = 0.067 [–0.015, 0.148], *p* = 0.111.

## Discussion

This study analyzed the relationship between levels of unmet needs in AYAs impacted by parental cancer and their psychosocial adjustment (i.e., health-related quality of life and internalizing problems) and explored the impact of demographic variables (offspring age and gender, and parental gender) and cancer variables (time since diagnosis and illness unpredictability) on level of unmet needs. In line with our first hypothesis, we found that higher unmet needs were negatively associated with health-related quality of life and positively related to internalizing problems. These results are consistent with previous studies indicating that parental cancer in AYAs is associated with higher unmet needs and distress [18, 20–22] and significantly impacts their psychosocial adjustment [8–10, 12, 13, 43]. We expanded these findings by highlighting an association not only with negative psychological outcomes but also with health-related quality of life which is a positive psychological outcome. This is, thus, the first study highlighting a negative relationship between unmet needs and health-related quality of life.

Furthermore, results of this study indicate that among demographics and parental cancer variables, only perceived illness unpredictability was significantly positively associated with offspring unmet needs. That is, higher levels of illness unpredictability were related to greater unmet needs in AYAs. These results are consistent with previous studies indicating that parental cancer relapse, which is related to the unpredictability of the illness, is associated with higher unmet needs in AYAs [20]. Also, results of this study might be explained by previous literature in which stronger subjective beliefs in AYAs toward the negative consequences of their parents’ illness were associated with the perception that the illness was unpredictable and with lower psychosocial adjustment, regardless of objective indicators of severity of parental illness [29]. The demographic and parental cancer variables included in our regression model explained 15% of the variance in offspring unmet needs, which indicates a medium effect size [44]. Other variables not assessed in this study might be also associated with offspring unmet needs. In fact, because parental cancer modifies family dynamics, decreasing the ability of the ill parent to fulfill familiar roles and responsibilities and increasing demands on other family members [28–30, 42, 45], AYAs might take on more caregiving responsibilities resulting in less time for social and activities and more unmet needs [46]. The quality of the relationship between family members might also affect the assumptions of caregiving responsibilities within the family and be associated as well with offspring unmet needs [29, 45]. Future studies should investigate how family role redistribution impacts on the levels of offspring unmet needs.

The second aim of this study was to examine the mediating role of unmet needs in the relationship between illness unpredictability and offspring psychosocial adjustment. A mediating effect of unmet needs was found between illness unpredictability and health-related quality of life. In particular, higher levels of illness unpredictability were related to higher levels of unmet needs which, in turn, were associated with lower levels of health-related quality of life. This finding is in line with the broader literature in which offspring of parents with cancer reported poorer quality of life as a result of parental cancer diagnosis [8–12, 47]. We clarified that lower levels of health-related quality of life were mediated by higher levels of offspring unmet needs due to greater illness unpredictability.

Unexpectedly, offspring unmet need did not mediate the relationship between illness unpredictability and internalizing problems. According to the broader parental cancer literature, AYAs of parents with cancer are at more risk of internalizing problems [14, 15] and offspring unmet needs were associated with higher levels of internalizing problems in this study. Nevertheless, we did not find that higher levels of internalizing problems were mediated by higher offspring unmet needs via higher levels of illness unpredictability. The lack of a significant mediation of offspring unmet needs in the link between illness unpredictability and internalizing problems might be explained by the fact that our sample had a mean parental time since diagnosis of three years while most other studies on the psychosocial adjustment of AYAs have been conducted with shorter mean parental time since diagnosis [8, 20]. Future research should further explore the association between illness unpredictability, offspring unmet needs, and internalizing problems in AYAs.

Results of this research highlighted perception of illness unpredictability as a factor implicated in psychosocial adjustment and unmet needs among AYAs over and above other demographic and cancer variables (i.e., offspring age and gender, parental gender, and time since diagnosis). Understanding this can assist in identifying at-risk AYAs with higher perception of parental illness unpredictability and unmet needs, and providing tailored interventions to improve their psychosocial adjustment to parental cancer. These interventions could include the following: (1) increasing AYAs understanding of, and active involvement in, their parents’ cancer treatment and care through psychoeducation and attending medical appointments with their ill parent [48]; (2) encouraging discussion about cancer within the family and the participation of AYAs in peer recreation/therapeutic camps which provide an opportunity for psychosocial support, skill development, and time out from the daily stresses of living with a parent affected by cancer [49]; and (3) using acceptance and commitment therapy based therapeutic approaches that facilitate having a different relationship with the realities of uncertainty and unpredictability while focusing the young person’s energy on living their life in line with their values [42, 50].

This study has several methodological limitations. First, the non-random sampling increases the risk of volunteer response bias and limits the generalizability of findings. However, participants were recruited from various cancer-related local community organizations as well as other recruitment strategies targeting youth (e.g., schools and youth groups) in order to obtain a sample as representative as possible of the Italian population of AYAs in the context of parental cancer. Nonetheless, our sample had an unbalanced distribution in terms of parents’ cancer type and gender of AYAs—as frequently reported in psycho-oncology research [51], there were over-representations of parents with breast cancer and of female AYAs. Furthermore, because of the cross-sectional design, inferences about causal directionality among illness unpredictability, offspring unmet needs and their psychosocial adjustment remain unclear. As mediation consists of causal processes that unfold over time, further research should examine longitudinally the mediational role of unmet need as a mechanism through which illness unpredictability exerts its effect on AYAs psychosocial adjustment.

## Conclusion

Our study has underlined that higher offspring unmet needs are associated with lower levels of health-related quality of life and internalizing problems and that higher levels of illness unpredictability are related to higher unmet needs. Finally, we showed that levels of unmet needs significantly mediated the relationship between illness unpredictability and offspring health-related quality of life (but not internalizing problems). These results enhance our understanding of offspring psychological adjustment by providing novel insight into the relationship between offspring unmet needs and positive (health-related quality of life) and negative (internalizing problems) offspring adjustment outcomes. Our findings also indicate that illness unpredictability is positively associated with offspring unmet needs and that it is the only significant predictor of unmet needs over and above demographics (gender, age, parental gender) and parental time since diagnosis. Perception of illness unpredictability may be a clinically relevant variable to target in supporting these young people given the greater risk of elevated levels of unmet needs and psychosocial difficulties.

## Data Availability

The data that support the findings of this study are available from the corresponding author upon reasonable request.

## Footnotes

1 Ancillary analyses showed that the strongest association between illness unpredictability and dimensions of offspring unmet needs were with dealing with feelings, “time out,” and recreation and support from other young people.

## Code availability

Codes of all analyses conducted in *Mplus* are available from the corresponding author upon reasonable request.
